# Perioperative Outcomes of Minimally Invasive Sacroilliac Joint Fusion Using Hollow Screws Through a Lateral Approach: A Single Surgeon Retrospective Cohort Study

**DOI:** 10.7759/cureus.16517

**Published:** 2021-07-20

**Authors:** Hamid Abbasi, Nick Storlie, Mitch Rusten

**Affiliations:** 1 Neurosurgery, Ambulatory Surgical Clinic, Tristate Brain and Spine Institute, Alexandria, USA; 2 Neurosurgery, Inspired Spine Health, Minneapolis, USA; 3 Research, Inspired Spine Health, Burnsville, USA

**Keywords:** sacro iliac joint, sacro iliac joint injections, sacro iliac fusion, chronic low back pain (clbp), atypical back pain

## Abstract

Sacroiliac joint (SIJ) pain is a common cause of lower back pain and a significant source of disability in the United States. There is no consensus on the best surgical treatment for SIJ pain that is not responsive to conservative therapy. Minimally invasive fusion of the SIJ using hollow fenestrated screws from a lateral trajectory is a newer technique for SIJ fusion. This study presents perioperative and patient-reported outcomes amongst 62 patients who underwent SIJ fixation with hollow fenestrated screws. We find that mean disability on the Oswestry disability index improved from 52.2% to 34.9% at one-year post-op. Mean operative time was 34±9 minutes and blood loss was 22±35ml. Only six patients required overnight hospitalization. There were two cases of complications requiring operative intervention. We conclude that SIJ fixation using hollow fenestrated screws is a safe and effective procedure for the fixation of the SIJ. Further investigation is warranted to determine the best surgical treatment for SIJ pain.

## Introduction

Chronic lower back pain (LBP) is one of the most common and debilitating conditions impacting patient populations, affecting 9.4% of people and contributing more than any other cause to disability globally [1]. This pain arises from various anatomical structures, most commonly the lumbar intervertebral discs, facet joints, and the sacroiliac joint (SIJ) [2]. SIJ pathology may be contributing to symptoms of up to 30% of all patients presenting with LBP [3] and an even larger share of patients with previous lumbar fusions [4]. The pain caused by SIJ dysfunction has been shown to impact the quality of life to an equal degree as more commonly recognized spinal pathologies such as disc herniation or spinal stenosis [5].

The sacroiliac joint is innervated by the lumbosacral nerve roots and is capable of minor physiologic movement and rotation despite being encased in strong ligaments [6]. Although the cause of SIJ pain is not fully understood, it is thought that abnormal motion of the joint results in inflammation and pain. Diagnosis of SIJ pain is made based on history and exam, as imaging studies can often be normal. Per medicare criteria, diagnosis of SIJ pain requires positivity on at least three out of the following six provocation tests: thigh thrust test, compression test, Gaenslen’s test, distraction test, Patrick’s sign, and posterior provocation test. Current treatments for SIJ pain aim to ablate the nociceptive nerves, reduce SIJ inflammation, or fuse the joint to prevent pathological movement [7]. Conservative treatments for SIJ dysfunction include physical therapy, therapeutic SI injections and radiofrequency ablation (RFA). While these interventions are useful as the first choice of treatment for SIJ dysfunction, there is mixed evidence for the long-term efficacy of both therapeutic injections and RFA [8]. SIJ fusion has become the standard treatment in patients with persistent SIJ pain that fails to respond to conservative measures.

Traditionally, SIJ fusion was performed using an open technique that required accessing the SI joint through a large incision with subsequent decortication of the joint, packing of bone graft, and fixation with screws or plates. Over time, minimally invasive approaches have become the standard of care for candidates of SIJ fusion due to the minimization of soft-tissue damage, reduced blood loss, lower operative times, and quicker recovery, with 85% of SIJ fusions being minimally invasive in 2012 [9]. There is no consensus on the preferred approach or technology for minimally invasive SIJ fusion, and several techniques are currently used [10]. One technique involves the percutaneous implantation of triangular titanium implants with the goal of transarticular stabilization and long-term biological fixation and fusion [11]. Another common type of fusion utilizes hollow or solid screws to achieve transarticular stabilization and promote fusion.

Here, we present data on a series of patients undergoing SIJ fixation using hollow fenestrated screws, placed transarticularly through a lateral incision with a trajectory perpendicular to the SIJ. We describe our technique for screw placement and report on one-year clinical outcomes of 62 patients who underwent SIJ fixation with hollow fenestrated screws.

## Materials and methods

Study Design

This study is a retrospective cohort study of 62 patients with SIJ disease who underwent 78 SIJ fixations. Procedures were performed by a single surgeon in 7 hospitals in Minnesota. Pearl Pathways IRB granted institutional review board (IRB) exemption. Inclusion criteria for this study were patients >18 years who underwent SIJ fixation between 1/1/2015 and 5/31/2020 by the study surgeon. Exclusion criteria were the presence of osteomyelitis, tumours, and the presence of severe lumbar pathology, traumatic injury before or after surgery, uncontrolled psychiatric comorbidities, and patients with possible secondary gain such as workers compensation. Patients who underwent unilateral SIJ fixation and underwent evaluation of the contralateral SIJ at the time of study conclusion were also excluded.

**Figure 1:**
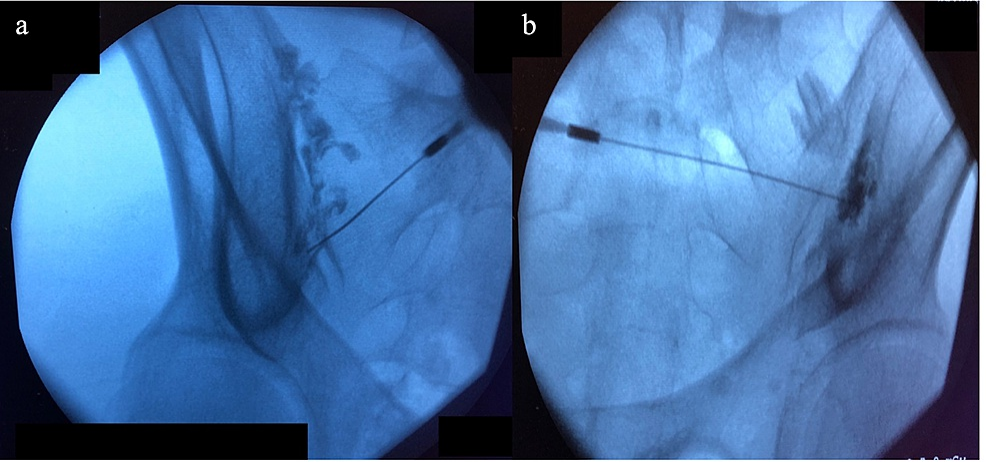
Diagnostic SIJ injection under (a) Illiac Crest View and (b) Outlet View Contrast can be seen inside the sacroiliac joint, confirming correct placement of needle.

Patients were candidates for surgery if they had SIJ pain diagnosed by the following clinical protocol. Clinical diagnostic criteria were a history of characteristic pain, typically unilateral caudal to the lumbar spine, and at least three positive provocation tests on clinical exam per Medicare criteria [12]. Radiculopathy patterns of pain can be produced by SIJ pathology and were not exclusion criteria for diagnosis of SIJ pathology. Patients who were clinically positive for SIJ pain were started on physical therapy and offered therapeutic and diagnostic SIJ injections. Diagnostic injections were performed using 2cc of 1% Bupivacaine with contrast confirmed placement (figure 1) and considered positive if patients reported at least 75% improvement of pain with diagnostic injections. Additional imaging, including CT of the lumbar spine and pelvis, myelogram, and MRI of the lumbar spine, was performed on a case-by-case basis to rule out alternative diagnoses but was not used to diagnose SIJ pathology. All patients underwent at least six months of conservative therapy, including physical therapy and at least three therapeutic injections using 40mg of Triamcinolone acetonide.

The SIJ Fixation Procedure

**Figure 2:**
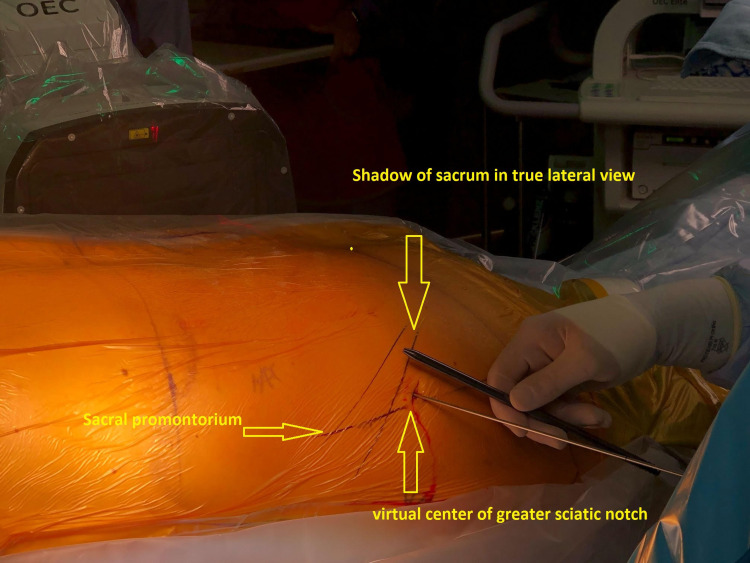
Marking and Incision Site The sacrum is marked in true lateral view. A line is drawn connecting the sacral promontory to the center of the greater sciatic notch. The incision is approximately one inch above the greater sciatic notch.

**Figure 3:**
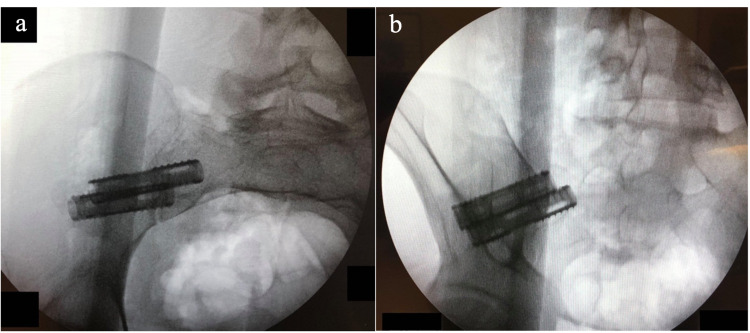
Completed SIJ Fusion Completed fusion of the SIJ under (a) AP and (b) Oblique fluoroscopic view. The screws seen are LNK hollow fenestrated screws.

We position the patient in the prone position and set up lateral C-arm fluoroscopy to visualize and align the greater sciatic notch and ala of the sacrum. We mark the sacral shadow on the skin and draw a line from the sacral promontory to the middle of the sciatic notch. The skin incision is made 1 inch perpendicularly above this line at the middle of the sciatic notch (figure 2). We infiltrate the local anaesthetic and enter the dull end of the guidewire until we make contact with the iliac crest so that it aligns with the S1-S2 foramen in the lateral view. We then stabilize the approach trajectory with a dilator and switch to the sharp end of the guidewire. We use this procedure to reduce the risk of vascular injury to branches of gluteal arteries. Once the entry point is established just cranial to the S1/S2 foramen, we use the pelvic inlet and outlet view to adjust the guidewire trajectory. The inlet view is the true axial view of S1/2/3, and the outlet view is the true AP view of the sacrum. We use these views to ensure the trajectory of the screws remains within the confines of the sacrum. We then insert the guidewire into the iliac crest and sacrum using the stryker battery-operated pin driver. Once the trajectory is confirmed in the inlet and outlet views, we perform serial dilations until the working cannula is inserted. We drill 0.5-1cm into the sacrum using the 6.5mm and 11.5mm drills. We collect the bone marrow extracted and mix it with biologic made from hydroxyapatite and tricalcium phosphate. We insert the screw and stimulate the screw until 30mA to rule out contact with neural structures. When using hollow screws, we pack the centre of the screw with the bone graft material. We remove the working portal and repeat the 1-1.5cm process more caudally to place the second screw. The completed fusion is seen under fluoroscopy in figure 3. During the study period, we used Zyga (Surgalign, Minnetonka, MN, USA), Corelink (Corelink LLC, St. Louis, MO, USA) and LnK screws (L& K Biomed Korea Inc., Seoul, Korea). The Zyga screw does not have a hollow interior cavity, so we used a curved curette to decorticate the internal surface of the SIJ to aid in fusion when using this screw.

Outcome Measures and Analysis

Skin to skin surgery time, blood loss, fluoroscopy time and hospital stay were recorded and entered into a custom database immediately after discharge. Because no suction is used, blood loss was measured by postoperatively weighing sponges and subtracting their dry weight. Patients underwent a physical examination and completed a modified Oswestry disability index (ODI)[13] before surgery and at the one-year follow-up, defined as having taken place at least 300 days after surgery to give patients flexibility in scheduling their appointments. For patients who had bilateral SIJ fusions performed separately, one-year follow up was defined as 300 days after the last operation. We recorded the following complications: bleeding, infection, vascular complication, and revisions. Data were collected in real-time, placed in a custom database and exported for analysis and visualization in R3.4.

## Results

Perioperative outcomes

Perioperative outcomes are in table 1. There were 78 operations involving 62 patients because 16 patients had a fusion of both SIJ in two separate operations. Mean operative time was 34±9 minutes and blood loss was 22±35ml. Overnight hospitalization was required for six operations (8.6%) for ambulation or pain management.

**Table 1:** Perioperative outcomes Perioperative outcomes of patients undergoing SIJ fusion Surgery times are skin to skin. SD: Standard Deviation; BMI: Body Mass Index

Operations (patients)	78
BMI (mean (SD))	33.4 (9.8)
Age (mean (SD))	54.0 (14.4)
Blood Loss (ml) (mean (SD))	22.2 (35.7)
Surgery Time (min) (mean (SD))	34.0 (8.5)
Fluoroscopy Time (s) (mean (SD))	169.5 (75.3)
Overnight Stay (%)	6 (8.6)

Patient-reported outcomes

Patient-reported outcomes are in Table 2. Preoperative Oswestry was available for 55 patients (87%), and post-operative Oswestry at the one-year follow up could be obtained for 44 patients (71%). Mean preoperative disability on the ODI was 52.2%, which improved to 34.9% at the one year follow up (p<0.001). Patients experienced significant improvements in each sub-category of the ODI.

**Table 2:** Patient-reported disability on the Oswestry disability index Table shows pre-op scores, post-op scores and p value obtained using two sided t-tests. To give patients flexibility in scheduling their appointments, the post-op appointment was defined as having taken place at least 300 days after surgery. SD: Standard Deviation

	Pre-op	Post-op	p
Number of Patients	55	44	
Pain (mean (SD))	3.2 (1.3)	2.1 (1.6)	<0.001
Care (mean (SD))	2.1 (1.3)	1.2 (1.4)	0.002
Lifting (mean (SD))	3.4 (1.0)	2.5 (1.6)	0.001
Walking (mean (SD))	2.8 (1.2)	2.0 (1.5)	0.003
Sitting (mean (SD))	2.1 (1.1)	1.5 (1.2)	0.013
Standing (mean (SD))	2.8 (1.1)	2.1 (1.5)	0.01
Sleeping (mean (SD))	2.4 (1.2)	1.3 (1.2)	<0.001
Social (mean (SD))	2.8 (1.6)	1.5 (1.4)	<0.001
Travelling (mean (SD))	2.1 (1.1)	1.3 (1.0)	0.001
Housework (mean (SD))	2.5 (1.1)	2.0 (1.4)	0.033
Score (mean (SD))	52.2 (16.9)	34.9 (21.1)	<0.001

Complications

One patient had a gluteal hematoma requiring evacuation and was later found to have an iliac artery pseudoaneurysm requiring coiling. Five patients were incidentally found to have minimal loosening of screws on imaging that did not require revision and did not correlate with clinical symptoms. One patient had hardware loosening requiring revision. One patient required placement of a central line because the anaesthesia team could not obtain peripheral access before the surgery. One patient experienced wound dehiscence without infection, which subsequently healed by secondary intention with local wound care.

## Discussion

SIJ fixation is an established treatment for SIJ pain that is not responsive to non-operative therapy. However, there is no consensus on the best approach to achieving surgical fixation of the SIJ. Here, we provide a case series adding to the literature on SIJ fixation using screws. We show that the procedure effectively improves patient-reported disability, with a 16-point decrease on the ODI one year after surgery. We also demonstrate that the procedure can routinely be performed as an outpatient procedure with surgery time well under an hour and only 6% of patients requiring an overnight admission.

The outcomes in our study are in line with other studies of SIJ fixation using screws. A previous study on 75 patients undergoing SIJ fusion using hollow modular anchorage screws found patients’ disability on the SF-36 physical health disability index improved from 42/100 to 26/100 [14]. This is a similar improvement as we report in our study, although improvements on the ODI and SF-36 cannot be compared directly.

Although several studies evaluate screws and plugs for a fusion of the SIJ, no studies directly compare different devices [1]. Studies involving plugs have generally documented significant improvements in patient-reported disability, with one randomized trial reporting a 24 point improvement on the ODI with a 4.7% revision rate [15]. The procedures took slightly longer (46 minutes) with more blood loss (51ml) than we reported in this study.

We favour the use of screws because we hypothesize that their stabilization mechanism provides various advantages over other technologies.12 Screws have increased pull-out strength over plugs [16]. While the insertion of bone plugs can distract the joint while passing through the intraarticular space, the presence of variable thread pitch on the hollow screws can reduce this space which may speed up fusion. We also hypothesize that using hollow, fenestrated screws with subsequent insertion of bone graft results in faster fusion. Further study is warranted as there is little evidence comparing different techniques for SIJ fusion. A randomized controlled trial could be useful in establishing a consensus method of SIJ fusion.

Limitations of this study include that this is a retrospective study, and results may be biased by imperfect follow-up. Additionally, our practice setting in several Minnesota hospitals may not be representative of other patient populations, making it impossible to draw direct comparisons to other published studies. Finally, this study did not include a control group, meaning no conclusions can be drawn about the relative efficacy of the procedure compared to other treatment modalities and other surgical approaches.

## Conclusions

Minimally invasive SIJ fusion using hollow screws through a lateral to the medial trajectory is a safe and effective procedure in patients with SIJ pain who have failed conservative therapy. This procedure can routinely be performed on an outpatient basis, and patients report significant improvements in their Oswestry disability scores one year after the procedure. Given the significant burden caused by SIJ pain, more research is warranted to determine the best surgical treatment.
